# Supplementary material for: Closing the Gaps to Understand the Tick Transmission of Anaplasma marginale among Giant Anteaters (Myrmecophaga tridactyla) in Argentina
Source: Pathogens. 2020 Dec 9;9(12):1033. doi: 10.3390/pathogens9121033 (PMC7763026; doi:10.3390/pathogens9121033)
Supplement: Supplementary file 1 [file pathogens-09-01033-s001.pdf]

Table S1. Summary of the date and location in which each giant anteater was sampled. The last column shows the result for *A. marginale* molecular identification.

Giant Anteater	Year of Blood Sampling	Location	*A. marginale* Detection
GA 1	2011	El Socorro	Negative
GA 2	2013	El Socorro	Negative
GA 3	2013	San Cayetano	Negative
GA 4	2013	San Cayetano	Negative
GA 5	2013	San Cayetano	Positive
GA 6	2013	San Cayetano	Positive
GA 7	2013	San Cayetano	Negative
GA 8	2013	El Socorro	Negative
GA 9	2014	San Cayetano	Positive
GA 10	2014	El Socorro	Positive
GA 11	2014	San Cayetano	Positive
GA 12	2014	San Cayetano	Positive
GA 13	2014	San Cayetano	Positive
GA 14	2014	El Socorro	Positive
GA 15	2014	San Cayetano	Negative
GA 16	2014	San Cayetano	Positive
GA 17	2014	San Cayetano	Negative
GA 18	2016	San Alonso	Positive
GA 19	2015	San Alonso	Positive
GA 20	2015	San Alonso	Negative
GA 21	2015	El Socorro	Positive
GA 22	2015	San Cayetano	Negative
GA 23	2015	San Alonso	Negative
GA 24	2013	San Cayetano	Positive
GA 25	2016	San Cayetano	Positive
GA 26	2016	San Cayetano	Negative
GA 27	2016	San Cayetano	Negative
GA 28	2016	San Cayetano	Negative
GA 29	2016	San Cayetano	Negative
GA 30	2017	San Alonso	Positive
GA 31	2016	San Alonso	Positive
GA 32	2016	San Cayetano	Positive
GA 33	2016	San Alonso	Positive
GA 34	2016	San Alonso	Positive
GA 35	2017	Don Pablo	Positive
GA 36	2017	Don Pablo	Positive
GA 37	2017	Don Pablo	Positive
GA 38	2007	El Socorro	Positive
GA 39	2007	San Cayetano	Negative
GA 40	2007	El Socorro	Positive
GA 41	2007	San Cayetano	Negative
GA 42	2017	San Alonso	---
GA 43	2009	San Cayetano	Positive
GA 44	2010	San Cayetano	Negative
GA 45	2010	San Cayetano	Negative
GA 46	2009	San Cayetano	Negative
GA 47	2013	El Socorro	Negative
GA 48	2010	San Cayetano	Negative
GA 49	2010	San Cayetano	Negative
GA 50	2018	San Cayetano	Negative
GA 51	2018	San Cayetano	Negative

GA: giant anteater. --- No blood sample analyzed.
